
JOURNAL INFORMATION
==============================
NLM Title Abbreviation: ScientificWorldJournal
Journal ID: ScientificWorldJournal
NLM Journal ID: 101131163
Journal ID: TSWJ

The Scientific World Journal

ISSN: 2356-6140
EISSN: 1537-744X
Publisher: Wiley

ARTICLE INFORMATION
==============================
PMCID: PMC6083993
PMID: 30147463
DOI: 10.1100/tsw.2001.153
Article ID: 169709
Article version: 1
Subjects: Short Report

Developing a New Method for Detecting Islet Apoptosis

Al-Abdullah I. H.
Cabrera O.
Inverardi L.
Pileggi A.
Pugliese A.
Ricordi C.
Diabetes Research Institute, University of Miami School of Medicine, P.O.BOX 016960(R134), Miami, FL 33101, USA
Publication date: 2001
Electronic publication date: 2001 Oct 3
Volume: 1
First page: 32
Last page: 32
Copyright: Copyright © 2001 I. H. Al-Abdullah et al.
Copyright year: 2001
License: This is an open access article distributed under the Creative Commons Attribution License, which permits unrestricted use, distribution, and reproduction in any medium, provided the original work is properly cited.
License URL: https://creativecommons.org/licenses/by/3.0/

==============================
Miami *Nature Biotechnology* Short Reports
*TheScientificWorld* (2001) 1(S3), 32SR
ISSN 1532-2246; DOI 10.1100/tsw.2001.153

### DEVELOPING A NEW METHOD FOR DETECTING ISLET APOPTOSIS

Al-Abdullah, I.H.*, Cabrera, O., Inverardi, L., Pileggi, A., Pugliese, A., and Ricordi, C.
Diabetes Research Institute, University of Miami School of Medicine, P.O.BOX 016960(R134), Miami, FL 33101, USA
* iabdullah@med.miami.edu

**INTRODUCTION.** Current islet cell isolation procedures can only retrieve a portion of the total number of islets present in the pancreas. One factor affecting islet yield is a variable level of apoptosis that is associated with mechanical and chemical isolation procedures. A quick and reliable method to assess apoptosis would be therefore useful to determine the overall quality of final islet preparations. We have tested PhiPhiLux, a recently developed method for early detection of apoptosis, in isolated pancreatic islets.

**METHOD**. Human (n = 3) and porcine (n = 2) islets were isolated by the automated method and density gradients purification (1). Aliquots of highly purified islets (>95%) were cultured at 37°C and 5% CO2 in CMRL medium (Human) and complete TC199 media (Porcine). Viability and apoptosis were assessed within 24 hr of isolation (fresh islets) and after 7-14 days culture. Viability was assessed using fluorescein diacetate and propidium iodide (FDA/PI) staining. PhiPhiLux (OncoImmunin, Inc) is a fluorescent Caspase substrate more specific for caspase 3 like activities. It is uptaken by live cells and can be used to detect early apoptotic events in cell lines and lymphocyte populations (2). The fluorescent signal is similar to that of FITC. 200 IEQ from the fresh and cultured cells were stained with PhiPhiLux (50 µl and 10% FCS) for 45 min at 37°C, washed and mounted with an antifade reagent (Molecular probes) and examined using a fluorescence microscope. The entire staining procedure takes about 90 minutes.

**RESULTS.** Islet viability as assessed by FDA/PI staining appeared to be very well preserved in both fresh and cultured islets (90-95%). Subjective evaluation of the PhiPhiLux staining revealed that less than 1% of fresh human and porcine islet preparations stained. Among cultured islets, PhiPhiLux stained approximately 5% and 10% of porcine islets after culture for 7 and 14 days, respectively, while approximately 2% of human islets were stained after 7 days or more.

**DISCUSSION.** Our results show that PhiPhiLux can be successfully used to assess apoptosis in fresh and cultured pancreatic islets. This method is rapid and simple providing a valuable alternative to more cumbersome methods, such as TUNEL and Annexin. Very limited number of islet cells was apoptotic after isolation using the methods reported here, but increased apoptosis was detected in cultured porcine islets (5-10%). The ability to detect apoptosis in human and porcine pancreatic islets with this method must be further verified by comparing PhiPhiLux staining with more conventional apoptosis detection methods (i.e., TUNEL and annexin staining). This preliminary report simply illustrates the technical feasibility of a recently described approach to detect apoptosis in islets. If validated by further experiments, this rapid apoptosis detection method could be of assistance to evaluate pancreatic islet preparations before their use in research and transplant applications.

**REFERENCES.**

1. Ricordi, C., Lacy, P.E., and Scharp, D.W. (1989) Diabetes 38(Suppl. 1), 140-142
2. Komoriya, A., Packard, B.Z., Brown, M.J., Wu, M.-L., and Henkart, P.A. (2000) J. Exp. Med. 191, 1819-1828
